# Supplementary material for: Are carbon emissions trading and green financial instruments synergistic? -Comprehensive quantitative research based on content analysis
Source: PLoS One. 2024 Mar 7;19(3):e0298601. doi: 10.1371/journal.pone.0298601 (PMC10919601; doi:10.1371/journal.pone.0298601)
Supplement: S1 Data — (ZIP) [file pone.0298601.s001.zip › Data/地级市Z_F工作报告.zip!/░▓╗╒/╗┤▒▒╩╨/└┤╫╘╣1⁄2╓┌║┼í╛┬φ┐╦╩2╛▌═°í┐_2010.docx]

各位代表:
     现在，我代表市人民政府向大会报告工作，请予审议，并请市政协各位委员和其他列席人员提出意见。
 一、2009年工作回顾
      刚刚过去的一年，是新世纪以来我市经济发展最为困难的一年。面对金融危机的严重冲击，在省委、省政府和市委的坚强领导下，在市人大、市政协的监督支持下，市政府团结带领全市人民，认真贯彻党的十七大和十七届三中、四中全会精神，深入学习实践科学发展观，大力实施城市转型，坚定不移地推进工业化、城市化、城乡一体化，着力保增长、保民生、保稳定，全面完成市十四届人大二次会议确定的各项目标任务。预计全年实现生产总值375亿元，比上年增长11%；财政收入52亿元，增长22.6%；规模以上工业企业增加值195亿元，增长13.6%；固定资产投资280亿元，增长36.8%；社会消费品零售总额105.7亿元，增长18.5%；外贸进出口总额1.2亿美元，增长10%；城镇居民人均可支配收入13700元，增长7%；农民人均纯收入4600元，增长12%。经过积极争取，我市被国务院列入第二批资源枯竭型城市，2009年获得国家财力性转移支付2.41亿元。在全国首次城市公共文明指数测评中，我市位居地级城市第29名，并第三次蝉联全国创建文明城市工作先进城市称号。在全省首次“科学发展观指数”排名中，我市名列第6位。
     （一）工业经济企稳回升，结构调整扎实推进。积极开展服务企业年活动，认真落实保增长的各项政策措施，争取和落实扶持资金1.4亿元，减免涉企税费1.7亿元，向困难企业发放岗位和培训补贴1亿元。积极帮助中小企业解决融资难问题，市担保公司注册资本金追加到1亿元，8家小额贷款公司获批、3家挂牌营业。分行业、分区域开展了8次政银企对接活动，落实贷款6.3亿元。预计2009年，全市银行业金融机构各项贷款余额256亿元，较年初增加80亿元；中小企业贷款余额突破100亿元，较年初增加28亿元。大力推进产业结构调整，食品工业、机械制造、纺织服装、陶瓷建材等非煤产业加快发展，对工业增长的贡献率达到 73.5%，食品工业跃升为第二大主导产业。县区规模工业完成增加值72.2亿元，增长55%，占全市规模工业增加值的比重比上年提高12个百分点。企业规模不断壮大，全年新增规模工业企业119 家，年销售收入亿元以上企业超过50家，淮北矿业、皖北煤电位居中国企业500强第245名和437名。加快以企业为主体的技术创新体系建设，117个技改项目竣工，新增国家高新技术企业3家、省级创新型试点企业3家，高新技术产业增加值突破10亿元。
     （二）农村经济稳定发展，一体化试点步伐坚实。全面落实强农惠农政策，发放各类补贴2.4亿元。克服50年不遇重大旱灾的不利影响，粮食生产再获丰收。新建百亩以上蔬菜基地23个，新建扩建规模养殖小区（场）292个，蔬菜总产增长10%，肉蛋奶总产增长6%。大力推进农业产业化，新增规模以上农产品加工企业12家，新发展农民专业合作组织93个，近三分之二的农户加入农业产业化经营体系。不断提升农业综合生产能力，新增节水灌溉面积3.5万亩，改善除涝面积4.5万亩。完成华家湖水库除险加固工程。绿色长廊二期工程建设完成109公里，新建、完善农田林网12.3万亩，人工造林2.5万亩。扎实开展城乡一体化试点，市财政筹措1亿元引导资金用于一体化建设。完成5个试点镇（办）空间布局、产业布局、基础设施建设规划和集中居住区（中心村）建设方案。全市开工建设城乡一体化项目268个，建成191个；14个村民集中居住区全面开工，工程完成后可集中安置农民8.8万人，置换土地1.3万亩，拉动社会投资10多亿元。大力推进农村土地承包经营权流转，新增土地流转面积5.8万亩。实施农村清洁工程，人居环境进一步改善。
     （三）东进南扩成效显著，生态城市建设加快。围绕建设“双百双宜”城市，组织实施38个城市基础设施项目，完成投资14.2亿元。城市路网进一步完善，萧淮路淮北至百善段改建、五宋路改线和长山南路、栗苑路、张寨北路、董庄东路等骨干道路建成通车，合徐高速公路淮北连接线、人民东路、沱河东路、烈青路、方安路、栗苑东路开工建设，34.8平方公里的东部新区初具雏形，城市未来发展框架基本确立。严格依法有序实施城市拆迁改造，完成拆迁面积82.4万平方米。淮北火车站改造、市图书馆、市广电中心、市公安局应急指挥中心建设进展顺利，220千伏凤凰山输变电站投入运营，城区燃气管网改造和天然气置换全部完成，防汛排水系统进一步完善。房地产业快速回升，竣工房屋建筑面积130万平方米，销售面积160万平方米。组织编制淮北市生态城市建设规划，完成采煤沉陷区水资源利用与湿地修复规划，成功举办2009中国（淮北）国际生态城市建设论坛，对淮海路、人民东路、长山路等道路及广场进行绿化改造，完成东部荒山绿化1万亩，总投资4.8亿元的水网工程正式开工。规范城区地名，对13条城区道路进行命名、更名，街巷门牌正在分区设置。
     （四）“双百工程”扎实推进，投资保持高位运行。抢抓中央扩大内需和支持城市转型的政策机遇，大力推进重点项目“双百工程”建设，完成投资128.1亿元，新开工项目59个，竣工项目30个。工业投资力度不断加大，刘店煤矿、临涣焦化一期甲醇项目、临涣选煤厂扩建工程等项目建成投产，临涣综合利用电厂一期工程全部完工并投入运营，淮水北调管道输水工程建成通水。口子工业园暨万吨优质白酒扩建工程基本竣工，部分设施投入使用。天然焦项目主井竣工，宝迪二期肉鸡加工项目部分设备安装调试。杨柳、青东、袁一煤矿建设进展顺利，香港锦泰食品一期、索谷电缆一期、沪光电气一期、银丰铝业汽车配件生产线、安徽山河装备产业园等项目加快推进，粗苯加氢精制、南坪精细化工配套项目邹庄煤矿、龙盟碳酸钙造纸等项目开工建设。大唐淮北虎山电厂、平山电厂正在积极争取国家发改委核准。基础设施建设力度不断加大，泗永路改建超额完成年度任务，泗许高速公路淮北段开工建设。市政府与安徽电信公司、安徽移动公司签订战略合作框架协议，未来五年投入不少于15亿元资金，推进数字淮北建设。
     （五）商贸物流健康发展，消费市场稳中趋旺。出台《加快服务业发展的实施意见》，市财政设立专项扶持资金1000万元，重点支持商贸物流业发展。28个重点商贸物流项目完成投资22亿元。淮北汽车城建设进展顺利，煤机博览城三期主体完工，韩国商贸城奠基。投资近亿元的百路物流年业务收入超过1.5亿元。翔宇物流、蓝宇物流、市粮食物流中心加快建设。完成惠黎、海宫、黎苑等菜市场改造，全市第一家标准化室内菜市场相城农贸综合市场开业。成功举办 “好日子”大型商品博览会、首届“农超对接”洽谈会和“佳节购物季”活动。第三届房地产展示交易会成交住宅2852套、面积29.6万平方米，成交额6.3亿元。2009中国（淮北）煤矿机械博览会成果丰硕，贸易成交额17.3亿元，签约投资项目45个，总投资53.3亿元。大力拓展农村消费市场，继续实施“万村千乡”市场工程，新建农家店300家。积极实施“家电下乡”工程，兑付补贴资金2350万元。
     （六）改革开放持续深入，发展活力明显增强。市属106家经营服务性事业单位转企改制、市工商系统市场管办脱钩全面完成。在义务教育学校实施绩效工资，1.47万名教职工基础性绩效工资、5280名退休人员生活补贴发放到位。市、县区政府机构改革积极推进，基层医药卫生体制综合改革试点已经启动。农村金融产品和服务方式创新试点工作稳步展开。不断完善国有资产监管体系，行政事业单位经营性资产纳入监管范围。非公经济较快发展，新增私营企业951家、个体工商户1.1万户、吸纳就业2.3万人。大力推进企业招商、产业招商和以商招商，积极开展与央企对接活动，成功举办2009海外华商安徽行暨淮北产业对接会，组团参加中博会、第六届中国———东盟博览会等招商活动。
      全年引进市外资金188.5亿元,增长25.3%;实际利用外资1.3亿美元,增长27.7%;新建亿元以上项目14个;太平洋集团、博世集团、江苏开元等13家国际、国内500强或国内行业100强企业落户淮北。进一步加强园区功能建设,完成投资5.2亿元。市开发区龙湖项目区二期加快推进,3.3万平方米标准化厂房基本建成,台湾唯一、三九医药等企业落地。濉溪经济开发区不断壮大,杜集区机械制造产业加速集聚,烈山区宝迪食品工业园快速发展,相山区凤凰山经济开发区获省政府批准筹建。淮北商检顺利开检。
     (七)民生工程落到实处,人民生活持续改善。投入资金8.1亿元,重点实施了28项民生工程。生活保障类项目扎实推进。全年新增城镇就业人员3.3万人,城镇登记失业率控制在3.9%以内。4.5万名企业退休人员人均月增养老金109元。发放农村低保金和五保户供养资金4363万元。落实城市低收入住房困难家庭保障补助政策,发放廉租住房租赁补贴420万元。开工建设6312套廉租住房,滨河花园一期工程等经济适用房项目基本竣工。向1.2万名贫困重度残疾人发放了补助金。教育培训类项目有序开展。投入资金1.6亿元,落实义务教育改革政策,受益学生30万名。资助中等职业教育家庭贫困学生1780万元。超额完成新型农民培训和农民工技能培训任务。医疗卫生类项目稳步实施。提高新农合筹资标准和补偿封顶线,补偿总额1.2亿元。实施免费婚检,落实农村孕产妇分娩补助政策,对艾滋病、结核病等重大传染病人实行免费救治。建成乡镇卫生院2所、村卫生室66所、社区卫生服务机构24所。城镇居民医疗保险全面推进,参保人数52万人;城镇职工基本医疗保险统筹基金最高支付限额提高到5万元,大额医疗救助基金最高支付限额提高到20万元。基础设施类项目进程加快。解决10.3万农村人口饮水安全问题。建成10所农村敬老院。农村公路“村村通”工程全部竣工,提前一年实现“十一五”规划目标。农村文化建设类项目进展顺利。完成农村广播电视“村村通”工程,建成64个农家书屋、10个乡镇综合文化站、21个农民体育健身工程。
     (八)社会事业全面发展,社会大局安定和谐。深入实施科教兴市战略,12个科技项目列入国家和省科技计划,高新技术产业增加值增幅居全省首位。义务教育质量不断提高,职业教育取得突破性进展,6000亩教育园区完成选址,在全省率先组建区域性现代农业职教集团、机电类和服务业职教集团,以及2家小学义务教育集团。市实验高级中学新校区顺利开工,安徽矿业职业技术学院完成组建并开始招生。落实教师补充与优化长效机制,公开招聘录用教师404名。完成农村义务教育债务清理化解工作,清偿债务1.02亿元。实施中小学校舍安全工程,加固、重建校舍16.1万平方米。市人民医院病房大楼即将竣工,市中医院病房大楼建设进展顺利,甲型H1N1流感等突发传染病防控工作扎实有效。成功举办“祝福祖国”国庆60周年大型活动和第五届中国大运河文化节暨大运河保护与申遗高峰论坛,荣获全国未成年人思想道德建设工作先进城市称号。群众体育活动蓬勃开展,竞技体育取得新突破,我市运动员在第十一届全运会上获得金银铜牌各一枚。严格落实耕地保护制度,土地利用总体规划修编加快推进,城乡建设用地增减挂钩和建设用地置换工作有效开展。刘桥矿、临涣选煤厂和濉溪县等三个污水处理厂相继建成,节水型社会建设试点顺利通过中期评估,公共机构节能工作取得明显成效。预计全市万元生产总值能耗下降4.6%;化学需氧量减少5.48%,提前一年完成“十一五”减排任务,二氧化硫排放量减少9.1%。人口计生工作稳中有进,人口增长控制在省下达指标以内。残疾人事业加快发展,妇女儿童合法权益得到保障。积极开展“五五”普法和法律援助,社区换届工作顺利完成。统计服务水平进一步提高,第二次经济普查工作获全国先进。民族宗教、食品药品安全工作得到加强,外事侨务、防震减灾、人防、气象、档案、保密、对台等工作取得新成绩。配合省军区成功组织遂行多样化军事行动应急救援演练,双拥模范城创建工作顺利通过省考核验收。
      切实加强公共突发事件应急管理工作,全力维护社会和谐稳定。深入开展“信访积案化解年”活动,财政安排专款,设立了信访救助专项资金、涉诉执行救助专项资金,实行领导包案制度,全年化解积案50多件,信访工作保持全省先进位次。积极推进“安全生产年”活动,坚持每月开展一项重点领域专项整治,有效防止重特大恶性事故发生,全年事故起数和死亡人数实现双下降。进一步加强平安淮北、法治淮北建设,严厉打击各类刑事犯罪,切实解决群众反映强烈的热点难点问题,人民群众安全感和满意度不断增强。在2008年杜集区被省委、省政府命名为“平安县区”的基础上,2009年濉溪县、烈山区又获此殊荣。
      在过去的一年里,我们注重加强政府自身建设。围绕推进科学发展、加快城市转型,扎实开展深入学习实践科学发展观活动,着力解决一批群众反映的突出问题,基本实现了党员干部受教育、科学发展上水平、人民群众得实惠的总要求。坚持依法行政,制定并落实了《淮北市征收集体所有土地办法》、《关于构建淮北市行政审批绿色通道实施意见》等27项制度。自觉接受人大及其常委会的法律监督、工作监督和人民政协的民主监督,重视工会、共青团、妇联等人民团体意见,全年办理省、市人大代表议案和建议100件、政协委员提案166件。规范行政执法行为,出台并实施《关于淮北市规范行政处罚自由裁量权工作实施意见》,推行“企业生产宁静日”制度,深入开展民主评议机关活动;公务办公网实现市、县区和大部分乡镇公务资源共享,电子政务建设继续走在全省前列;行政服务中心审批网与部门自有审批网完成对接,实现远程审批,行政审批电子监察实现全覆盖,服务效能不断提升。完善目标管理体系,注重主要工作目标的强制约束、过程监督和结果运用,促进争先创优。加强惩治和预防腐败体系建设,在政府投资工程建设领域实行项目廉政保证金制度,加大政府投资工程招投标监管力度,强化行政监察和审计监督,深入开展“小金库”专项治理,廉政建设和反腐败工作取得新成效。
     各位代表!过去的一年,在世界金融危机冲击、各种困难集中显现的情况下,淮北在转型中发展、在逆势中奋进,经济社会发展好于预期,极为不易。这是全市上下共克时艰、奋力拼搏的结果,凝聚着全市人民的心血和智慧,得益于社会各界的大力支持和帮助。在此,我代表市人民政府,向全市广大工人、农民、知识分子、干部和离退休老同志,向各民主党派、各人民团体和社会各界人士,向驻淮解放军指战员、武警官兵和政法干警,向中央和省驻淮单位,向所有关心和支持淮北发展的朋友们,表示衷心的感谢并致以崇高的敬意!
      回顾过去的一年,我们清醒地认识到,全市经济社会发展还存在着一些突出矛盾和问题。从产业结构看,在经济周期性波动,特别是在金融危机的大背景下,产业结构的单一性表现更为突出,煤电产业仍占规模工业增加值的60%以上,现代服务业发展不足。从发展方式看,增长方式还比较粗放,产业链比较短,产品附加值比较低,部分企业经营困难。从发展动力看,县区和园区经济总量不大,非公经济发展不足,经济外向度偏低,经济企稳回升的基础还不稳固。从发展协调性看,城乡一体化工作刚刚起步,城乡居民收入差距较大,农村基础设施建设相对滞后,教育、文化、卫生等社会事业发展仍需加强。从政府自身看,职能转变还不到位,与人民群众的期待还存在一定差距,行政效能需进一步提高。对这些问题,我们将高度重视并认真加以解决。
    二、2010年主要任务
     今年全市经济发展环境总体向好。国内经济企稳回升,煤炭、电力等能源行业进一步回暖。《促进中部地区崛起规划》颁布实施,省委、省政府支持城市转型力度加大,为我市加快发展带来新的机遇。随着我市工业化、城市化、城乡一体化深入推进,一批重大项目相继建成,新的经济增长点正在加速形成,支撑经济平稳较快发展的动力和活力进一步增强。同时也应看到,当前世界金融危机余波未尽,国内经济回升还存在不确定不稳定因素,我市新上项目难度加大,自身发展还面临不少困难。我们必须坚定信心,增强忧患意识,做好应对各种困难和挑战的准备,把经济企稳回升的势头保持好、巩固好,在发展中促进转型,在转型中加快发展。
     2010年政府工作的总体要求是:全面贯彻党的十七大、十七届三中、四中全会和中央经济工作会议精神,以邓小平理论和“三个代表”重要思想为指导,深入学习实践科学发展观,调整结构促转型,加大投入增后劲,坚定不移地推进工业化、城市化、城乡一体化,大力实施重点项目“双百工程”、新兴产业培育工程、惠民利民工程,加快建设创新型、生态型、和谐型城市,奋力推动经济社会平稳较快发展。
     综合考虑各种因素,2010年全市经济社会发展的主要预期目标是:地区生产总值增长12%以上;财政收入确保突破60亿元大关,增长14%以上;规模以上工业增加值增长16%;固定资产投资增长25%以上;社会消费品零售总额增长18%;外贸进出口总额增长10%;城镇居民人均可支配收入增长8%;农民人均纯收入增长10%;城镇登记失业率控制在4.2%以内;万元生产总值能耗下降4.5 %;化学需氧量、二氧化硫排放量分别控制在1.46万吨和5.57万吨;人口增长控制在省下达指标以内。
     各位代表,预期目标是我们深入分析发展形势、紧密结合淮北实际提出的。平稳增长是基本要求,较快发展是努力方向。为实现上述目标,我们将切实抓好九个方面的工作:
     (一)坚定不移实施工业强市战略,着力增强工业支撑作用,推动经济持续增长。
     加大服务力度,推动工业经济较快增长。继续完善并落实好一系列扶持措施,全面支持,突出重点,强化对企业的服务。大力实施中小企业成长工程,采取补助、贴息、奖励等措施,鼓励和支持中小企业上规模、规模企业上台阶。支持金融企业创新产品和融资方式,增加对中小企业贷款,确保4家小额贷款公司运营。建立产品推荐目录,继续组织企业参加各类产品促销活动,力争全市工业产品销售率达到98%以上。
     加快结构调整,培育发展新兴产业。煤电等主导产业是我市的优势所在,也是稳定增长的基础支撑。继续推进传统产业升级改造,做大做强优势企业,做精做细优势产品,延长产业链,提高附加值,壮大产业总量和企业体量,充分发挥主导产业对全市经济增长的引擎作用。编制实施好新兴产业发展规划,研究制定培育新兴产业发展扶持政策,设立1亿元专项资金,重点支持科技含量高、成长性好、对经济发展能产生重大推动作用的项目,加快培育精细化工、新材料、生物医药、生物质能源等新兴产业,推进产业转型升级。支持华润三九中药产业园和秸秆造浆项目建设,中润生物公司尽快实现工业化生产,碳酸钙造纸一期工程上半年建成投产,力普拉斯二期项目力争年内竣工。
     推进自主创新,着力转变发展方式。坚持把提高自主创新能力、调整优化产业结构摆在更加突出位置,继续深化科技体制改革,加强科技创新支撑体系建设,整合建设精细化工科技企业孵化器,建设凤凰山专利(食品)创业园孵化器,发展和完善机电装备专业科技企业孵化器。规划建设科技创新公共服务中心,充分发挥综合科技服务平台作用。加强与科研院所合作,积极引进吸收高新技术,组织实施科技创新项目35个、技术改造项目230个,大力发展高附加值食品,加快煤矿机械制造等优势产业技术改造与研发生产,新增省级高新技术产品10个以上。进一步加大节能减排力度,突出抓好重点行业、重点企业和重点领域节能降耗,推行清洁生产。组织编制低碳经济发展规划,争取国家和省政策支持。大力开展植树造林,发挥碳汇减排功能。
     (二)远谋近施促进城乡统筹发展,继续稳固农业基础地位,加快城乡一体化进程。
     优化区域布局,促进高效农业规模化发展。进一步推动濉溪县国家级农业标准化示范区和省级现代农业示范区建设,深入开展小麦高产攻关活动和玉米振兴计划,突出优质高产高效,稳定发展粮食生产。继续落实果蔬基地、养殖小区建设和农业产业化发展、土地流转等奖补政策,培育1个万亩标准化设施蔬菜栽培基地、1个万亩标准化瓜果栽培基地、30个标准化规模养殖小区(场),培育一批“一村一品”示范村。
     发展壮大食品工业,推进农业产业化经营。研究编制农副产品加工业发展规划,打造食品工业高地。发挥宝迪、雨润、鲁王等龙头企业带动作用,确保口子工业园万吨优质白酒生产线、香港锦泰食品、福润禽类加工等项目竣工投产,力争天宏食品、六和勤强二期等项目尽快建成,力促杭州食品产业园、康宏园食品、浩大淀粉等项目按计划施工。加快濉溪县黑糯玉米基地建设,延长黑糯玉米深加工链条,支持淮北普豪生物科技公司尽快上马黑色素提取项目。做好宝迪肠衣加工项目前期工作。围绕龙头企业,大力发展规模养殖。加大农民专业合作组织的扶持培育力度,新发展农业合作经济组织69家。继续扶持培育专业大户等规模经营主体,年内新增土地流转面积3万亩。
     加强农田水利基本建设,提高农业综合生产能力。从今年开始,3—5年投资1.9亿元,建立较为完善的农田灌溉、除涝体系,探索建立农田水利工程运行长效机制。年内投资3765万元,新增、恢复有效灌溉面积18万亩,新增、改善除涝面积8万亩。加强重点工程建设,完成北淝河洼地治理工程和临涣闸扫尾工程。建设高标准农田林网8万亩,全面提升中南部农田林网建设水平。
     积极推进制度创新,抓好城乡一体化试点工作。市财政继续投入1亿元用于城乡一体化建设。在巩固5个试点镇建设成果的基础上,结合“扩权强镇”和工业园区建设,扩点强面。4月底前完成全市小城镇和集中居住区规划编制,加强村庄整治和小城镇、集中居住区建设,优化农村人居环境。推进农村土地整治,改善城乡用地结构,为建设用地提供保障。改革户籍管理制度,加快农民变市民步伐。开展集体土地上房屋确权登记工作,变资产为资本,活跃农村经济。创新经营体制,支持农民开展资金互助和土地股份合作。加快开放式村部建设,投资3718万元,新建、改扩建尚未达标的147个村级组织服务用房,6月底前全部建成并投入使用。进一步加大对农村教育、就业、社会保障、卫生、文化等方面的投入,促进城乡公共资源均衡配置。
     (三)千方百计拓展城乡消费需求,切实提升消费拉动作用,繁荣发展商贸服务业。
     加快重点商贸物流项目建设。继续抓好淮海时代广场、中泰广场、相王国际、和协国际等续建项目建设,开工建设润恒物流中心、濉溪影都等项目,做好张寨商贸项目、金鼎大厦项目等前期工作。建成大型农贸市场凤凰山农贸城,建成四星级酒店天赐良缘、中泰广场酒店;力争年内大润发、沃尔玛大型超市和金鹰国际大型商场开业。
     着力提高商贸流通发展水平。引入现代管理和连锁经营模式,推进商贸流通业向规范化、产业化发展。统筹区域内旅游景点建设,突出发展休闲旅游业。推进家政服务体系建设与早餐工程建设,完善社区服务设施,拓展服务领域。继续实施“家电下乡”和“万村千乡”市场工程,编制并实施中心城区菜市场布局规划,新建标准化菜市场2家、改造提升3家。继续推进“新网工程”,努力打造农村现代流通服务网络体系。加大“双百市场工程”扶持力度,支持中瑞等大型农产品批发市场建设。
     积极推进重点商贸区域建设。改造老城区商业网点,完善功能,提升档次和整体形象。大力引进国内外知名商业企业,注重大型连锁超市合理设点布局,推动商业网点品牌建设,提升新城区商贸服务功能。充分挖掘和利用资源,打造特色商业街区。加快淮北汽车城建设,发展新型建筑装饰材料等专业市场,培育产地型特色农产品市场,不断完善市场体系。抓好国家级商务综合执法试点工作,建立12312市场投诉举报、咨询服务平台。
     (四)持之以恒推进城市东进南扩,不断完善老城区服务功能,加快建设生态城市。
     加强城乡规划引导,严格城乡建设管理。编制村镇规划,逐步建立适应城乡统筹发展的村镇规划管理体制。启动2011—2015年城市近期建设规划编制工作,科学编制东部新城规划、城市综合交通规划和排水工程专项规划,完善产业园区规划。加强规划监管,维护城乡规划的严肃性、权威性,全方位提高城镇化发展水平。
     突出东部新区开发,全面拉开城市框架。加快建设东部新区 “五横五纵” 主干路网,确保合徐高速公路连接线、沱河东路3月底建成通车,加快方安路、淮海东路、董庄路东段、梅苑路东段、长山南路南段、新湖路、流河路等道路建设和宿丁路改造。推动水、电、气、污水管网、垃圾处理等基础设施建设,完善东部新区功能。围绕构建四区联动的宜业新城和山水交融的生态新城,建设以淮北煤师院、淮北职业技术学院新校区为重点的教育园区,以华家湖生态园区、东山生态休闲等为重点的旅游区。全力支持淮北矿业集团办公新区建设。探索城市优质公共资源向东部新区转移的新途径,逐步推进商务区和居住区建设。实施采煤沉陷区水资源利用与湿地修复规划,重点建设“55321”工程,修复构建生态水网,6月底前完成水网规划一期主体工程并下闸蓄水;加强湿地保护,凸显山水生态城市特色。积极构建绿地系统,加快东湖景区和南湖湿地公园建设,抓好东部山场绿化二期工程,新增荒山绿化1.67万亩。同时进一步推动相濉、相杜、相烈同城化。
     加快老城区改造,完善综合服务功能。推进淮北火车站和“城中村”改造,搞好城市供水、供气、亮化、污水管网等配套设施建设,适时推动城市集中供暖。完善城市防洪除涝工程,打通长山路沟,完成老铁路沟改建工程,沟通城市水系。加强淮海路、相山路、人民路、孟山路等主要街道景观整治,做好龙山路、淮海西路、北山巷和渠沟路等街巷整治改造。投资4000万元,按照国家4A级标准建设相山公园风景区,加快桓谭公园建设。不以追求土地收益、而以适应城市发展要求和提升市民宜居宜业水平为目标,严格拆迁程序,规范拆迁行为,切实保障被拆迁人的合法权益。
     推进文明城市建设,提升城乡文明程度。积极争创第二届全省文明城市和全国无障碍建设城市,进一步夯实创建全国文明城市工作基础。完善城市管理,构建城市管理长效机制。大力开展“清洁乡村、美化家园”活动,加大以城带乡、矿镇共建力度,提升农村创建工作水平,打造我市文明创建新品牌。从今年开始,将“清洁乡村、美化家园”的部分费用纳入市财政预算。
     (五)抢抓机遇加大投资力度,切实加快重点项目建设,进一步增强经济发展后劲。
     扎实推进重点项目建设。继续以重点项目“双百工程”为抓手,在抓投入中优化产业结构,在上项目中提升产业层次,确保有效投入增速不减。做大做强煤电产业,加快推进杨柳矿、青东矿、袁一矿、邹庄矿等项目建设,争取大唐虎山电厂尽快开工。不断壮大煤化工产业,加快8万吨粗苯加氢精制、30万吨煤焦油加工、鸿源煤化工二期项目建设,着力形成煤—焦—化—电产业链。扶持发展机械制造业,力争安徽山河矿业装备产业园一期工程4月份试生产,着力构建煤矿机械制造产业基地。加快振兴纺织服装业,力促唯一服饰等项目建设,着力形成纺纱—织造—印染—服饰配套产业链。改造升级陶瓷建材业,重点发展高中档陶瓷产品,培育钢构业和新型建材业。加快建设500千伏濉溪变电站扩建工程、110千伏滂汪输变电工程和泗许高速公路淮北段等基础设施项目。落实政府性投资计划,研究制定促进民间投资的具体措施,努力形成政府投资引导、社会投资主导的良性循环。
     积极做好项目前期工作和重点项目谋划。加强临涣煤焦化二期、南坪精细化工园、临涣综合利用电厂二期等重大项目前期工作。围绕城市转型,把握国家产业政策导向,结合编制“十二五”规划,精心谋划一批产业层次高、技术含量高、产品附加值高,能迅速形成规模效应、具有重大带动作用的大项目。在煤炭深加工、煤层气开发、煤矸石综合利用、煤焦油综合利用、高岭土深加工等方面谋划一批产业链延伸项目,在节能环保、“三农”、改善民生等方面对接一批国家重点支持的项目,在新兴产业、现代服务业、重大基础设施等方面,高起点谋划争取一批项目,进一步充实项目库,优化项目结构。
     (六)一以贯之促进县区和园区发展,大力提高产业集聚度,着力打造新的经济增长极。
     继续支持县区加快发展。牢固树立县区发展就是全市发展的重要理念,把强县强区作为全市经济社会发展的重要战略和基础来定位,坚持不懈地加以支持。扎实开展“扩权强镇”试点工作,下放部分行政审批权和执法权,简化项目审批环节。充分考虑试点镇发展需要,合理布局,统筹安排,在建设用地方面给予支持。继续加大对县区支柱产业、重点企业、重大项目的支持力度,进一步提高县区经济发展的质量和水平。 
     强力推进园区建设。投资9100万元,加快市开发区路网、污水处理及配套管网工程等基础设施建设。实施龙湖工业园东向发展战略,加快二期建设。依托市建投集团帮助县区融资,专项用于园区建设,进一步提升配套服务功能。逐步探讨建立园区准入门槛,引导产业向园区集聚,关联企业向特色园区集中,加快相山区凤凰山食品工业、烈山区和濉溪县百善农副产品加工、杜集区机械制造等产业集聚区建设,努力把园区建设成为定位差异化、集聚规模化、产业高新化、效益最大化、服务优质化、环境和谐化的增长极。
     (七)毫不动摇深化改革开放,培育壮大非公经济,不断激活发展动力。
     加快重点领域改革。全面推进医药卫生体制改革,抓好濉溪县、相山区综合改革试点,在政府举办的基层医疗卫生机构实行药品零差率销售补贴,逐步落实基本公共卫生服务均等化。继续推进相关事业单位绩效工资制度改革,逐步形成合理的分配激励机制。推进政法经费保障体制改革,提高保障水平。大力推进全民创业,加强创业基地建设,积极开展创业培训、创业辅导,综合运用税收、财政贴息和政府奖励等政策,鼓励和支持非公经济加快发展。坚持权利、义务和责任相统一,管资产和管人、管事相结合,进一步加强国资监管,实现国有资产保值增值。
     加大招商引资力度。立足互利双赢,进一步加强与央企、与大企业对接,着力引进产业带动强、科技含量高、投资强度大、资源消耗低的好项目。创新招商方式,积极开展产业招商和大项目定点招商,突出企业招商、以商招商,在选商补链上下功夫。办好2010中国(淮北)煤矿机械博览会,精心筹划一批重大招商引资活动,争取引进世界500强和国内500强企业取得新突破。改善投资环境,落实优惠政策,加强签约项目跟踪服务,完善招商引资激励约束机制、重点项目服务机制,不断提高招商项目履约率、资金到位率和项目开工率。全年利用内资235亿元、外资1.56亿美元,均增长25 %以上。
     努力推动出口稳定增长。充分利用进出口备案登记权下放我市的有利条件,壮大外贸增长源,鼓励更多企业开展自营进出口业务,扩大进出口规模,优化进出口结构。同时,落实与南京、徐州等城市合作框架协议,加强区域产业、科技、人才交流与合作。 
     (八)更加注重优化财政收支结构,扩大有效信贷投放,努力增强服务发展的能力。
     进一步做好财税工作。坚持开源和挖潜并重,加强重点行业和重点税种征管,堵塞征管漏洞,保证各项税收及时足额入库。强化支出管理,细化部门预算,提高资金使用效率。实行公务用车“核定编制、统一管理”制度,因公出国(境)计划经费联合审核制度,会议定点接待制度,以及支出绩效考核制度,推行公务卡管理制度,实现公务支出零增长或负增长。扩大政府采购范围,规范政府采购行为。全面启动财政科学化精细化管理,提高财政管理水平。　
     促进金融支持经济发展。加强政银企合作,出台并落实降低企业融资成本实施意见,组织银行业金融机构帮扶中小企业。完善信用担保体系,深化金融生态环境建设。大力推进农村银行、村镇银行、农村资金互助社等新型农村金融机构组建试点工作,力争年内引进1—2家商业性银行,鼓励支持县区和市开发区设立小额贷款公司。继续培育企业上市资源,进一步拓宽融资渠道,优化投融资结构。积极做好政策性农业保险工作。
     (九)扎实有效实施惠民利民工程,坚持不懈发展各项社会事业,加快构建和谐淮北。
     全面落实改善民生的各项政策措施,持续加大投入力度,实施好33项民生工程,进一步加强社会建设。
     以人为本解民忧,努力提高社会满意度。继续实施积极的就业政策,深化创业型城市创建工作,大力促进高校毕业生就业,加强弱势困难就业群体就业援助,开展农村劳动力技能培训和“阳光工程”培训,统筹推进城乡就业,全年新增城镇就业岗位3万个,下岗失业人员再就业7000人,帮扶就业困难人员再就业2000人,新增农村劳动力转移就业8000人。进一步推进义务教育均衡发展,巩固扩大素质教育成果。弘扬高尚师德,打造高素质教师队伍。继续实施中小学校舍安全工程,确保完成3年改造加固任务的90%以上。抓好小区建设配建教育设施工作,缓解主城区教育资源紧张状况。规划市一中新校区,力争市实验高中新校区建设基本完工。支持淮北煤师院更名和淮北职业技术学院申报省级示范高职学院。加强农产品加工、养殖、商贸物流等职业教育培训,为新兴产业发展提供技能人才支撑。
     践行宗旨谋民利,努力提高社会幸福度。完善社会保障体系,做好企业退休人员基本养老金调整工作,探索建立农村新型社会养老保险制度,落实城乡社会救助政策。提高新农合人均筹资标准,统一区、县报销补偿标准。做好甲型H1N1流感等传染病防控和突发公共卫生事件处置工作。加快市社会福利中心、老年公寓及康复设施与敬老院建设,开工建设市救助管理站和儿保中心,确保市中医院病房大楼投入使用。多方筹资6亿元,建设廉租住房20万平方米、经济适用住房20万平方米。落实强农惠农政策,做好粮食直补、良种补贴、农机具购置补贴、农资综合直补工作。
     恪尽职守保民安,努力提高社会安全度。认真落实安全生产责任制,加强安全生产隐患排查和治理,坚决遏制重特大事故发生,确保安全生产形势持续稳定好转。进一步完善防控体系,强化社会治安综合治理,加强重点地区治安整治,严厉打击黑恶势力和各类违法犯罪活动,做好网络监管工作。巩固全市食品药品专项整治成果,建立和完善食品药品安全监管体系,健全长效监管机制。高度重视并妥善处理群众来信来访,加强人民调解工作。完善应急管理工作机制,提高突发公共事件应急处置能力,切实维护社会稳定。
     群策群力促民和,努力提高社会文明度。做好庆祝建市50周年各项工作,加快重点献礼工程建设,纵深推进城乡环境综合整治,让全市人民共享发展成果,进一步焕发建设新淮北的巨大热情。继续繁荣发展文化事业,扶持发展文化产业。加强文化市场管理,严肃查处非法出版和传播不良文化等违法违规行为,落实文化市场管理长效机制。做好第二届全市道德模范评选和第二届全省道德模范推荐工作。推进中国大运河保护与申遗工作和柳孜遗址二期考古发掘,确保市图书馆、市广电大楼投入使用,国家矿山公园3月底全部完工。大力开展全民健身运动,不断丰富人民群众体育文化生活,积极备战省第十二届运动会,市新体育场4月份开馆。继续实施土地整理复垦开发,探索建设用地置换指标有偿调剂,完成土地利用总体规划修编工作。加强矿产资源管理,做好城市地质调查工作。深化环保专项行动,严格环境执法监管,加快烈山区、市开发区、濉溪开发区、临涣工业园污水处理厂和杜集区污水管网建设。积极迎接水利部对我市节水型社会建设工作的全面验收。继续推进公共机构节能工作。加强人口和计划生育工作,完善计划生育利益导向机制,综合治理出生人口性别比偏高问题,基本实现农村计生工作“村为主”。强化价格监管。认真做好第六次全国人口普查。抓好外事侨务和对台事务。做好防震减灾、气象、档案、地方志、机关事务管理等工作。重视和支持妇女、老龄和残疾人事业,加强未成年人保护。积极发展社会福利事业和慈善事业。切实做好民族宗教工作。加强国防教育和人防工作,大力支持驻淮部队建设,提升双拥工作水平,巩固军政军民团结。
    三、进一步加强政府自身建设
     全面完成今年的各项目标任务,必须切实抓好政府自身建设,不断提高行政服务水平。我们将以科学发展观为指导,牢固树立正确的政绩观,把更多的精力用在深入基层调查研究上,用在解决热点难点问题上,用在为群众办实事办好事上,用在打基础、管长远的工作落实上,努力做到对人民负责、受人民监督、让人民满意。
     (一)坚持创新创造创优,解放思想促发展。继续深入开展学习实践科学发展观活动,着力抓好整改落实,不断完善体制机制,巩固扩大学习实践活动成果,进一步增强政府工作人员落实科学发展观的自觉性和坚定性。始终保持兢兢业业、争创一流的拼搏精神,直面挑战、迎难而上的进取精神,勇于突破、敢为人先的创新精神,认真学习借鉴先进经验,用新办法解决新问题,用新机制激发新活力。认真总结“十一五”规划实施情况,深入研究改革发展中的重大问题,科学编制“十二五”发展规划,以新视角谋划新思路,以新举措创造新优势。
     (二)坚持科学民主阳光,依法行政重规范。全面完成“五五”普法规划,进一步完善科学民主的决策机制。自觉接受人大及其常委会的法律监督、工作监督和人民政协的民主监督,认真办理人大代表议案、建议和政协委员提案。广泛听取各民主党派、工商联、无党派人士与各人民团体的意见,自觉接受社会公众和新闻舆论监督。强化执法监督,做好行政复议工作,严肃整治行政不作为、乱作为。继续深化行政审批制度改革,推进行政审批规范化管理,构建绿色通道。推进政务公开和服务承诺,实施阳光听证、阳光审批、阳光监督、阳光问责,保障人民群众的知情权、参与权、表达权、监督权,不断提高政府的公信力。
     (三)坚持求实务实落实,勤勉理政干事业。积极倡导雷厉风行、奋发有为的工作作风,建立健全高效快捷、迅速果断的执行机制,做到有事即议、议则早定、定则快办、办则办好。大力精减会议、精减文件,严格控制和规范各类检查、评比、验收活动,重实际,说实话,求实效。进一步优化政府组织结构,简化行政流程。继续加强电子政务建设,实现全市政务信息互联互通和资源共享,推动机关业务网上运行。完善目标管理与政务督查联动机制,加大重点工作过程监督和奖惩力度,确保政令畅通。深入推进诚信政府建设,做到承诺有度、承诺有信、承诺有效。
     (四)坚持教育制度监督并重,清正廉洁固本色。落实廉政建设责任制,深入推进惩治和预防腐败体系建设,加强对政府性投资项目建设及资金监管,从源头上防范工程建设领域腐败现象。坚决纠正损害群众利益的不正之风,着力解决涉及群众利益的突出问题,完善权力运行的监督制约机制,认真查处各类违法违纪案件。时刻牢记“两个务必”,大力发扬艰苦奋斗、勤俭节约的优良传统,把有限的资金和资源更多地用在发展经济、改善民生上,做勤政廉洁的人民公仆,树风清气正的政府形象。
    各位代表,今年是实施“十一五”规划的最后一年,也是为“十二五”发展奠定基础的关键之年。让我们在市委的坚强领导下,坚定信心不动摇,迎难而上不退缩,抢抓机遇不懈怠,求真务实不折腾,同心同德,负重拼搏,奋力推动经济社会平稳较快发展,以优异的成绩向建市50周年献礼!
